# Short-Term Memory Capacity Predicts Willingness to Expend Cognitive Effort for Reward

**DOI:** 10.1523/ENEURO.0068-24.2024

**Published:** 2024-06-28

**Authors:** Brandon J. Forys, Catharine A. Winstanley, Alan Kingstone, Rebecca M. Todd

**Affiliations:** ^1^Department of Psychology, University of British Columbia, Vancouver, British Columbia V6T 1Z4, Canada; ^2^Djavad Mowafaghian Centre for Brain Health, University of British Columbia, Vancouver, British Columbia V6T 1Z3, Canada

## Abstract

We must often decide whether the effort required for a task is worth the reward. Past rodent work suggests that willingness to deploy cognitive effort can be driven by individual differences in perceived reward value, depression, or chronic stress. However, many factors driving cognitive effort deployment—such as short-term memory ability—cannot easily be captured in rodents. Furthermore, we do not fully understand how individual differences in short-term memory ability, depression, chronic stress, and reward anticipation impact cognitive effort deployment for reward. Here, we examined whether these factors predict cognitive effort deployment for higher reward in an online visual short-term memory task. Undergraduate participants were grouped into high and low effort groups (*n*_HighEffort_ = 348, *n*_LowEffort_ = 81; *n*_Female_ = 332, *n*_Male_ = 92, *M*_Age_ = 20.37, *Range*_Age_ = 16–42) based on decisions in this task. After completing a monetary incentive task to measure reward anticipation, participants completed short-term memory task trials where they could choose to encode either fewer (low effort/reward) or more (high effort/reward) squares before reporting whether or not the color of a target square matched the square previously in that location. We found that only greater short-term memory ability predicted whether participants chose a much higher proportion of high versus low effort trials. Drift diffusion modeling showed that high effort group participants were more biased than low effort group participants toward selecting high effort trials. Our findings highlight the role of individual differences in cognitive effort ability in explaining cognitive effort deployment choices.

## Significance Statement

We must often make decisions about when cognitive effort is worth the potential reward. Reward value, depression, and chronic stress in rodents can impact cognitive effort deployment decisions for reward, but factors like short-term memory ability can only be easily characterized in humans. We examined whether short-term memory ability, depression, chronic stress, and reward anticipation predict cognitive effort decisions for reward. In a short-term visual memory task with a choice of easier or harder trials for low versus high reward, we found that only short-term memory ability predicted more choices of high versus low effort trials. This research suggests that cognitive effort decisions could be driven by cognitive effort ability more than motivational factors like depression or chronic stress.

## Introduction

We are often faced with difficult choices about work and life. For example, we may choose to spend time thoroughly studying for an exam to gain a few extra percent points on a grade; alternatively, we may trade off this reward to work less and be able to do other tasks. These choices require trading off more or less effort for a larger or smaller reward, and thus involve deciding how much cognitive effort to deploy. In general, the motivation to deploy cognitive effort can be influenced by the potential reward to be gained (Shenhav et al., 2013, 2016; Yee et al., 2021). Beyond this, an individual’s willingness to expend cognitive effort can also be linked to individual differences in factors that influence overall motivation, such as mood disorder levels (Grahek et al., 2019; Yee et al., 2019; Pruessner et al., 2020). Research in rodents has revealed patterns of individual differences in motivation to exert cognitive effort, where rats are classified as “workers” (high effort group) or “slackers” (low effort group) depending on whether they are willing to work more or less for reward, respectively (Hosking et al., 2016; Silveira et al., 2020, 2021). However, factors influencing individual differences in willingness to deploy cognitive effort for reward have yet to be fully characterized in humans.

In both rodents and humans, chronic stress (Birn et al., 2017; Watt et al., 2017) and depressive traits (Grahek et al., 2019; Silveira et al., 2020) have been shown to negatively impact cognitive effort deployment for reward. In rodents, chronic stress dampens reward anticipation even as acute stress heightens it (Ironside et al., 2018; Kúkel’ová et al., 2018). Here, chronic stress is typically induced by prolonged social defeat (Kúkel’ová et al., 2018) or non-social chronic mild stress, such as modifications to housing (Slattery and Cryan, 2017). In humans, chronic stress—unlike acute stress—cannot ethically be induced. Research into the impact of chronic stress on reward motivation primarily focuses on reports early childhood stress (Birn et al., 2017; Watt et al., 2017), and not shorter-term chronic stress of the kind that university students may experience (Towbes and Cohen, 1996). This latter form of chronic stress is more widely experienced than early childhood stress, and yet its impact on our willingness and ability to complete everyday cognitive tasks is not well understood.

Alongside chronic stress, depression is another motivational factor that has been shown to dampen reward anticipation (Westbrook et al., 2018, 2020; Terpstra et al., 2023). In particular, anhedonia—a key symptom of depression impacting interpretations of reward (Slattery and Cryan, 2017)—can drive reduced reward anticipation and negatively impact willingness to deploy effort in rodents (Scheggi et al., 2018). Similarly, in humans, trait-level anhedonia (Treadway et al., 2012, 2009) and anticipation of anhedonia (Sherdell et al., 2012) generally predict reduced motivation to deploy physical effort for reward (Culbreth et al., 2018). However, many tasks in life are cognitive as opposed to physical in nature; cognitive effort tasks may offer more naturalistic value as a result.

Decisions on when to deploy more or less cognitive effort require attentional control in both humans and rodents. Such decisions can be driven by executive processes such as visuospatial working memory (Engstrom et al., 2013). Greater working memory ability can, in turn, drive better attentional control (Unsworth and Robison, 2020). Furthermore, during cognitively effortful tasks, reward anticipation modulates activation in neural circuitry that supports working memory (Fuentes-Claramonte et al., 2015). Reductions in executive function capability—for example, given high levels of chronic stress and depressive traits—could drive excessive reliance on reward incentives, leading to maladaptive effort deployment (Kool et al., 2010; Sandra and Otto, 2018). Although visuospatial working memory tasks can be conducted in rodents, including those exhibiting chronic stress and depression phenotypes, cognitive factors impacting cognitive effort deployment in humans are more complex than those that we can evaluate with rodents (Stephan et al., 2019). Acknowledging and accounting for these fundamental differences between human and rodent experiences of the world is an important yet underexamined element of translational research. Therefore, it is important to translate rodent cognitive effort tasks (rCETs) into human tasks, allowing us to test human-based theories of cognitive and attentional control while maintaining comparability with rodent work.

In humans, the expected value of control theory (Shenhav et al., 2013) predicts that, in general, people will deploy more cognitive effort when they know that they can effectively obtain greater rewards by doing so (Frömer et al., 2021). Additionally, the theory predicts that greater reward anticipation will lead to a higher expected value of deploying cognitive effort to obtain a reward (Grahek et al., 2020). Past work suggests that dopaminergic processes motivate physical and cognitive effort deployment decisions to maximize rewards (Michely et al., 2020) and to weigh rewards more heavily than effort costs. These processes also interplay with serotonergic processes that promote reward learning and biasing people toward more cognitively effortful options (Westbrook et al., 2020) even when it is suboptimal to deploy effort earlier—which could occur more often in people with low levels of short-term working memory ability (Raghunath et al., 2021). However, the degree to which individual differences in reward anticipation and executive function capacity influence the likelihood of choosing to deploy more cognitive effort for higher reward is not known.

In rodents, different levels of motivation between “workers” and “slackers” have also been associated with differences in learning rate and bias toward choosing high effort trials, which may be linked to differences in cognitive capacity. Drift diffusion modeling (DDM) in rodents completing a cognitive effort task has revealed that “workers” with high accuracy may accumulate evidence more quickly toward selecting high effort, high reward (HR) trials than “slackers” or “workers” with low accuracy (Hales et al., 2024). In an equivalent task in humans, such evidence could include awareness of one’s effort deployment willingness and ability as well as fatigue. For example, participants may build experience with how the task demands align with their short-term memory capabilities, as well as their willingness to push these abilities further for higher reward. Overall, the above drift diffusion model findings suggest that effort cost computations could vary with a combination of effort demands and reward value as factors we rely on to make judgments about when to deploy more or less cognitive effort for reward. In turn, individual differences in reward anticipation may be linked to depressive traits or chronic stress, and rodent research indicates that executive capacity may also influence choice behavior (Eichenbaum, 2017). The goal of the present study was to examine the degree to which individual differences in reward anticipation, chronic stress levels, and depressive traits on the one hand, and visuospatial short-term memory as an index of executive function on the other, predict human choice behavior independently or in interaction.

### The present study

One way of examining factors that motivate humans to deploy more or less effort for reward is to offer participants the choice of completing an easier or harder cognitive effort task for a low or HR, respectively (Treadway et al., 2009). In the current study, we built on an existing rodent choice task to investigate whether visual short-term memory capacity, chronic stress, depressive traits, and reward anticipation predict one’s choices in deploying cognitive effort for reward. A variety of human tasks exist to evaluate the costs of effort decisions. Some of these tasks examine the point at which high versus low effort and reward options are equally preferred, including the cognitive effort discounting task (Westbrook et al., 2013; Crawford et al., 2021). In contrast, physical effort deployment tasks, such as the effort expenditure for rewards task (Treadway et al., 2009), typically offer a binary effort choice structure with an easy trial for a lower reward or a harder trial for a higher reward. A key aim of our study was to examine whether human cognitive effort decisions can be modeled based on established animal models of cognitive effort deployment (Cocker et al., 2012; Hosking et al., 2016; Silveira et al., 2021). Furthermore, the use of two effort levels encourages participants to strategize according to the trial difficulties available rather than fully discounting excessively easy or difficult trials offered in a parametric design. Thus, to ensure continuity with rCETs—including a binary choice paradigm—while maintaining a similar incentive structure to existing human cognitive effort tasks, we adapted the rCET (Cocker et al., 2012; Hosking et al., 2016; Silveira et al., 2020) for use in humans. In the rodent paradigm, rodents first learn to associate illuminated lights with the opportunity to obtain a reward. In the main phase of the task, rodents are given the choice between a low effort, low reward (LR) trial or a high effort, HR trial. Then, in a basic memory task, they must poke their noses in a hole that lights up for either 1000 ms (LR) or 200 ms (HR) (Cocker et al., 2012). Rodents must rapidly encode the location of the illuminated light to succeed. Such a task can be scaled up to human working memory capabilities by including more stimuli and features that must be encoded to obtain a reward. For our study, we used harder and easier conditions (larger vs smaller set size) from a visual short-term memory task (Luck and Vogel, 1997) to serve as high and low cognitive effort choices within the same kind of choice paradigm offered in the rCET. In each trial, participants could choose to perform an easier visual short-term memory task for a lower reward or a harder task for a higher reward. We examined overall visuospatial short-term memory capacity as well as indices of depressive traits (Beck et al., 1996), chronic stress (Levenstein et al., 1993), anhedonia (Rizvi et al., 2015), and reward anticipation (Terpstra et al., 2023) as predictors of the tendency to choose lower or higher effort tasks. Furthermore, we used DDM (Ratcliff, 1978; Hales et al., 2024) to examine factors that may contribute to choice biases.

## Materials and Methods

### Participants

We powered our study using a power analysis through the pwrss package (Bulus, 2023) to achieve an expected power of 80% at an odds ratio of 0.70 for a logistic regression evaluating the probability of a participant selecting 70% or more high effort trials (being in the high effort group in the study). This analysis gave us a target sample size of *N* = 487. We recruited *N* = 570 participants from the Human Subject Pool of psychology undergraduate students at the University of British Columbia; they were each compensated with bonus points for their courses as well as a CAD (Canadian dollar) $5.00 Starbucks gift card. Of these, n=42 participants did not complete the initial survey and n=7 participants did not complete the reward anticipation task. Furthermore, n=11 participants completed the change detection task more than once, n=2 participants spent more than 30 s choosing any one trial—as this could indicate disengagement with task demands, and n=79 participants performed at or below chance (50%) during the choice phase of the task. In total, we analyzed data from N=429 participants (n=92 male, n=332 female, n=5 other; Table 1). The study was approved by the Behavioural Research Ethics Board at the University of British Columbia, approval code H20-01388.

**Table 1.** Demographic information for all participants, by sex and effort deployment group

Group	Sex	*n*	*M*_age_	SD_age_	Min_age_	Max_age_	*M*_BDIprop_	SD_BDIprop_	*M*_PSS_	SD_PSS_	*M*_DARS_	SD_DARS_	*M*_ant_.	SD_ant_.
High effort	Female	263	20.34	2.37	16	42	0.27	0.18	20.83	6.21	35.61	11.71	3.94	1.81
	Male	80	20.70	2.34	17	32	0.19	0.14	17.69	5.30	37.69	9.62	4.29	1.75
	Other	5	20.40	0.89	19	21	0.17	0.10	19.60	3.65	43.00	10.07	4.80	1.64
Low effort	Female	69	20.01	1.94	16	26	0.28	0.16	20.68	6.54	38.04	8.94	4.00	1.79
	Male	12	20.92	2.97	18	28	0.19	0.14	21.08	4.38	39.17	8.61	3.96	1.51

BDI prop: beck depression inventory II proportion score (score divided by max score); PSS: perceived stress scale score; DARS: dimensional anhedonia rating scale; Ant.: mean reward anticipation in the behavioral monetary incentive delay task; high effort group: >70% HR trials selected; low effort group: ≤70% HR trials selected.

### Stimulus presentation

All participants completed the study online on their own devices, via the Pavlovia online study platform using PsychoPy 2021.2.3 (RRID: SCR_006571; Peirce et al., 2019). Participants were not allowed to complete the study on mobile phones or tablets.

### Stimuli

**Figure 1.**
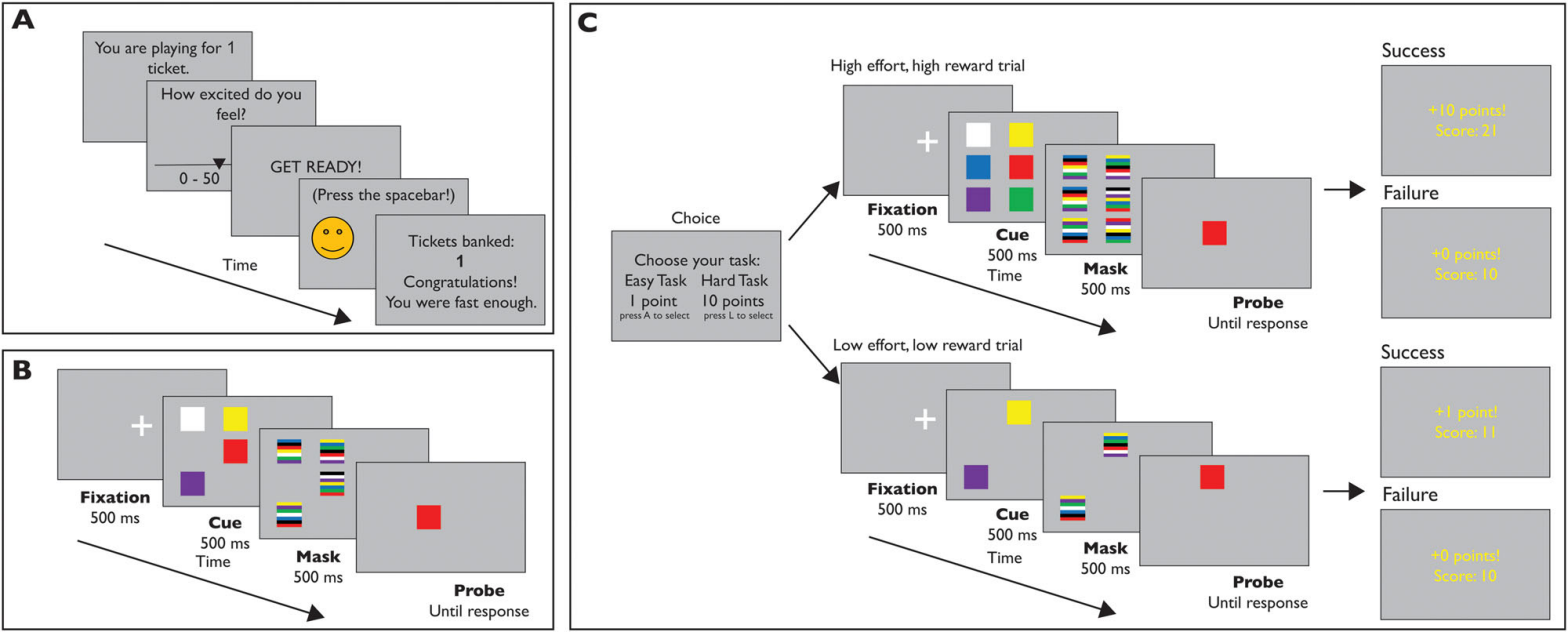
Layout of the experimental tasks. ***A***, The monetary incentive delay task, in which participants indicate their excitement in playing for tickets toward a monetary reward. ***B***, The calibration phase of the change detection task, where participants saw an array of either two, four, six, or eight squares and indicated whether the final square that appeared (the probe) was of the same or a different color from the square that appeared in the same location in the previously show array. ***C***, The reward phase of the change detection task, where participants saw an array of either two or six, or four or eight, shapes onscreen depending on their performance in the calibration phase of the task. Once again, participants indicated with a keyboard press whether the final square that appeared (the probe) is the same or a different color from the square that appeared in the same location in the array. Here, if they made the correct decision, they would receive 1 point (low effort trial) or 10 points (high effort trial); they would receive 0 points for an incorrect decision.

All stimuli used in the study were generated by and implemented in PsychoPy (Peirce et al., 2019). In the first phase of the study, participants performed an online behavioral monetary incentive delay task as a measure of reward anticipation (Fig. 1*A*) as outlined by Terpstra et al. (2023). In brief, during this initial task phase, participants saw a series of scales that they could click to indicate how excited they were to receive a reward. After a brief wait, participants would see a small happy face appear on one side of the screen. Afterward, they were shown whether they received a reward or not, and were asked to view another scale where they would indicate how excited they felt. They completed this task for a series of eight trials.

In the second phase of the study, as a measure of each individual’s visuospatial short-term memory capacity, we presented a visuospatial short-term memory task (Fig. 1*B*,*C*) modified from Luck and Vogel (1997) and adapted from an open-source version of the task on Pavlovia (de Oliveira, 2023). In this task, we presented a series of trials with between two and eight colored squares that were presented for 500 ms. Each square subtended a visual angle of approximately 0.05° on the screen. A mask with multi-colored squares would then appear at each of the original squares’ locations for another 500 ms, followed by a single colored square appearing in one of the positions of the original squares. This final square had a 50% chance of being the same or a different color from the square appearing in the same position in the initial part of the trial. After indicating with a keyboard press whether the square was the same or a different color from the initial square, they would see whether or not they gained points toward a monetary reward.

### Procedure

#### Questionnaires and monetary incentive delay task

Participants began the study by completing an online questionnaire. After giving consent and demographic information, they were asked how about their history of depression and anxiety, COVID-related stress, the perceived stress scale (PSS; Levenstein et al., 1993); the beck depression inventory II (BDI; Beck et al., 1996), and the dimensional anhedonia rating scale (DARS; Rizvi et al., 2015). Afterward, they were redirected to the first phase of our study, the online behavioral monetary incentive delay task (Fig. 1*A*; Terpstra et al., 2023). This task measured participants’ reward anticipation via the mean of the excitement ratings given before each trial.

#### Visuospatial short-term memory task

Versions of this task served both as a means to measure individual differences in visuospatial short-term memory as well as the tasks of varied cognitive effort to be subsequently chosen for high/low effort trials. After completing the monetary incentive delay task, participants were redirected to the second phase of our study, the visuospatial short-term memory task (Luck and Vogel, 1997). Here, this task served as a means to measure individual differences in visuospatial short-term memory as an index of working memory. After receiving instructions on which stimuli would appear and how to respond to them, participants first completed a series of 10 practice trials presented in a random order. In half of these trials, the probe square was the same color as that of the cue square (congruent/no change trial); on the other half, the probe square was a different color from that of the cued square (incongruent/change trial). Of these practice trials, four had a set size = 2, another four had a set size = 4, and another two had a set size = 6. In each trial, the probe square could be anywhere in the array, and participants were required to hold the whole array in visual short-term memory in order to successfully indicate whether the probe color had changed. Participants did not receive feedback on whether their responses were correct or not in the practice block, in keeping with Luck and Vogel (1997). Following these practice trials, participants completed (Fig. 1*B*) a series of 120 trials of the visuospatial short-term memory task with 60 change and 60 no change trials. In total, 30 trials of each set size (2, 4, 6, and 8 squares onscreen) were presented in a randomized order. Although the original task by Luck and Vogel (1997) contained trials with up to 10 squares onscreen, this largest set size was determined to be too difficult for participants to reliably complete correctly following piloting; as such, the maximum set size was eight. Furthermore, although the main task had a maximum set size of eight, the practice block only included a maximum set size of six so as to give a brief overview of the trials and required responses without giving excessive practice with a large set size, for which performance would be evaluated in the subsequent block.

Based on participants’ performance in this phase of the task, a K estimate of their visuospatial working memory capability (Rouder et al., 2011) was calculated for each set size, as follows:

$${\hat{k}}_{\;p}=N\left(\frac{\hat{h}-\hat{f}}{1-\hat{f}}\right),$$

where *N* is the set size, $\hat{h}$ is the hit rate, and $\hat{\;f}$ is the false alarm rate. As participants had to evaluate the whole display to judge the color of the resultant single probe (Rouder et al., 2011), we used a whole-display K estimate measure (Pashler, 1988).

In addition to serving as indices of individual differences in visuospatial short-term memory, K estimate scores were used to calibrate the tasks used in the effort choice task (Fig. 1*C*), which was based on the rCET (Hosking et al., 2016; Silveira et al., 2021). Here, participants completed a series of 30 trials. They would receive rewards for making a correct response, and they could choose a high effort, HR trial or a low effort, LR trial at each trial. Importantly in this task, trials with small and large arrays from the visuospatial short-term memory task served as the LR and HR trials. HR trials used a larger set size, but gave a reward of 10 points toward a monetary reward. LR trials had a smaller set size than the high effort/HR trials, but yielded a reward of one point toward a monetary reward. We set the high effort trial reward to 10 points to motivate participants to continue deploying effort even for a much more difficult task. In order to ensure that the task difficulty in this phase was balanced by participants’ working memory capability, we used participants’ K estimate score at set size = 4 from the initially presented visuospatial short-term memory task. This set size was determined through piloting to be the maximum set size before performance dropped off. Performance at set size = 4 was used to set a criterion for the available set sizes in HR and LR trials. Specifically, if the K estimate at set size four was ≤3, participants could choose an LR trial with a set size of two or an HR trial with a set size of six. If the K estimate at set size four was >3, participants could choose an LR trial with a set size of four or an HR trial with a set size of eight. Although participants were told that the number of points they gained in this phase was proportion to the monetary reward they would receive, all participants received the same reward (a $5 CAD Starbucks gift card) at the end of the study. Afterward, participants were redirected to a debriefing survey and received their course credit and monetary reward.

Our primary outcome measure of interest was the proportion of time participants chose high versus low effort trials. In order to conduct secondary analyses evaluating whether participants’ tendencies to choose high effort trials for HR explained performance (accuracy in the reward phase of the task) and choice latency (time until participants selected a difficulty level in the reward phase), we classified participants into two categories according to the criteria discussed by Silveira et al. (2021): participants who chose the HR option for more than 70% of all trials in the reward phase were in the high effort preference group, while participants who chose the HR option for less than or equal to 70% of all trials in the reward phase were in the low effort preference group. This grouping was chosen to ensure continuity with comparable cognitive effort studies in rodents (Hosking et al., 2016; Silveira et al., 2021; Hales et al., 2024). Although the grouping binarizes the outcomes of our analyses, it offers a clear point of translation from the rodent literature on which our task is based, and allows us to predict whether participants exhibit a high effort motivation versus low effort motivation phenotype.

### Analyses

All analyses were conducted using R 4.3.1 “Beagle Scouts” (R Core Team, 2023) through RStudio (Posit team, 2024).

The primary predictor variables in our study were (1) working memory ability, operationalized as a participant’s K estimate at a set size of four; (2) depressive traits, operationalized as a participant’s BDI proportion score; (3) chronic stress levels, operationalized as a participant’s PSS score; and (4) reward anticipation, operationalized as a participant’s mean excitement before playing for tickets in the monetary incentive delay task (Terpstra et al., 2023). As our overall depression score measures were of more translational interest than anhedonia, given the role of depression in downweighing effort deployment and reward anticipation in both rodents (Slattery and Cryan, 2017) and humans (Grahek et al., 2019), we focus on reporting this measure as opposed to the anhedonia measure we also collected. The primary dependent variable in our study was (1) proportion of HR trials chosen in the reward phase of the task, operationalized as whether participants chose the HR option for more or less than 70% of all trials in the reward phase. In additional analyses, we further examined the effects of willingness to expend high effort for HR on the following dependent variables in the rewarded visuospatial short-term memory task: (2) accuracy, operationalized as the proportion of correct responses (hits and correct rejections), and (3) choice latency, operationalized as the time in seconds until participants selected a difficulty level for each trial. To assess whether participants who consistently chose higher difficulty trials were overall better at the task compared to those who chose low difficulty trials, we compared accuracy between high and low effort groups. To examine whether, as predicted by Hales et al. (2024), choice latency was longer for all LR versus HR trials—as opposed to whether the tendency to choose high versus low effort trials explained how long participants took to make a decision when selecting a trial—we further compared choice latency between high and low effort groups. Finally, drift diffusion model parameters of drift rate, starting point, boundary separation, and non-decision time (Hales et al., 2024) during choices in the effort choice (reward) phase of the task were additional outcome variables.

For our analyses, we first conducted a series of *t* tests to evaluate sex differences in BDI and PSS scores. Past work suggests sex differences in depressive traits (Altemus et al., 2014; Forys et al., 2023) and chronic stress levels (Watt et al., 2017), with women presenting with higher depression and chronic stress scores than men. For sex difference comparisons, only male and female participants were included as we were under-powered to report sex differences from those reporting sex as “other”. As participants may express high levels of both depressive traits and chronic stress—potentially impacting reward anticipation (Alloy et al., 2016)—we also evaluated the extent to which BDI scores, PSS scores, and reward anticipation were correlated with each other. Next, we divided participants into high and low effort groups to ensure translational comparability with existing rodent work. As by Cocker et al. (2012), participants who chose the HR option for more than 70% of all trials in the reward phase of the task were in the high effort preference group, while those who chose the HR option for 70% or less of all trials in this phase were in the low effort preference group. We first conducted a binomial linear regression to determine whether sex, working memory ability, depressive traits, chronic stress levels, and reward anticipation significantly predicted whether a participant was in the high or low effort group in terms of their effort choices. As choices for high effort trials may also vary continuously with visual short-term memory ability, we then conducted a linear regression to evaluate whether the above regressors also predict the proportion of high effort trials selected. We then conducted two 2 × 2 within-between ANOVAs (trial type × group) using anova_test through the rstatix package (Kassambara, 2023) to evaluate whether accuracy or choice latency significantly differed by trial effort level (HR vs LR) and motivation group (high effort vs low effort groups). We examined accuracy on each trial and choice latency for selecting the trial difficulty to evaluate whether participants were matched for performance and time spent selecting a trial (Cocker et al., 2012), regardless of how many high versus low effort trials they selected. We then ran two multi-level models through the lmerTest package (Kuznetsova et al., 2017) to evaluate whether sex, working memory ability, depressive traits, chronic stress levels, reward anticipation, and effort level significantly predict choice latency or accuracy.

Lastly, in order to evaluate whether the high and low effort groups differed in response strategies and biases toward selecting HR versus LR trials, we fit a hierarchical Bayesian drift diffusion model, adapted from Ratcliff (1978) and run for 2,000 iterations with 1,000 warmup iterations and four Markov chains for Monte Carlo sampling, to data from all participants using the hBayesDM package in R (Ahn et al., 2017). We used this model to compare drift rate, starting point, boundary separation, and non-decision time between participants who chose high levels of effort versus those who chose low levels of effort. The drift rate captures the rate at which participants drift toward a decision boundary (selecting an HR or LR trial). The starting point is expressed as a percentage of the distance between the lower and upper boundaries; it captures the initial bias, at the beginning of each trial, that participants have toward selecting an HR over an LR trial. The boundary separation, expressed relative to zero, captures a tradeoff between speed and selecting the HR trial as opposed to the LR trial. Lastly, non-decision time captures the part of choice latency that is not related to cognitive effort decisions, such as the time required to execute a motor response upon making a decision.

## Results

### Depressive traits and chronic stress levels

**Figure 2.**
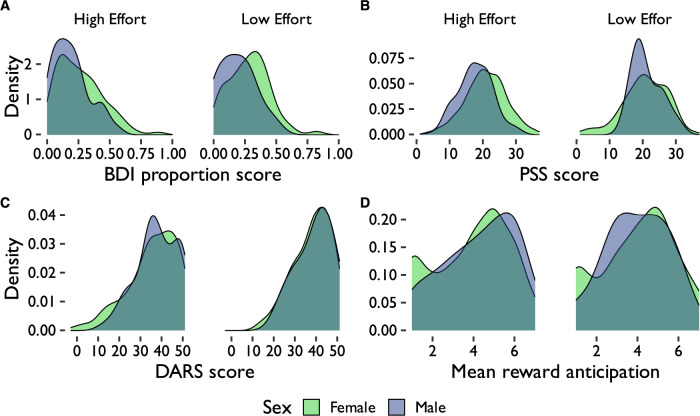
Distribution of ***A***, depressive trait (BDI) proportion scores (score divided by total possible score), ***B***, perceived stress (PSS) scores, ***C***, anhedonia (DARS) scores, and ***D***, mean reward anticipation scores by sex and effort deployment group. BDI, beck depression inventory; PSS, perceived stress scale; DARS, dimensional anhedonia rating scale.

**Figure 3.**
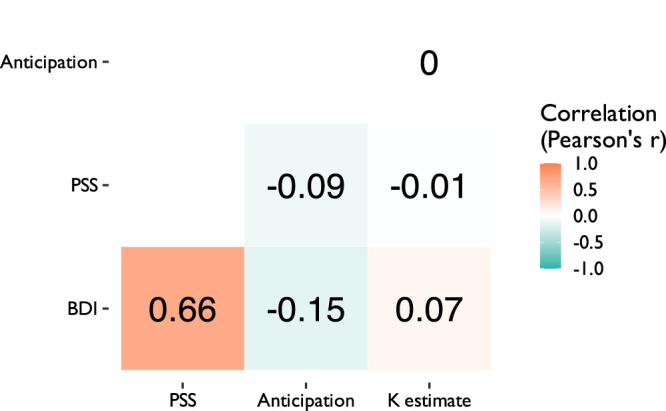
Correlations among depressive traits (BDI), perceived stress (PSS), reward anticipation, and short-term memory ability (K estimate). BDI, beck depression inventory; PSS, perceived stress sale; anticipation, mean reward anticipation; K estimate, visual short-term memory ability (K score).

Summary statistics for depressive traits (BDI) and chronic stress levels (PSS) can be found in Table 1. After correcting for multiple comparisons using the Bonferroni method, women had significantly higher depressive trait scores (t(261.23)=5.35,p≤0.001,d=0.45; Fig. 2*A*) and chronic stress scores (t(270.03)=4.93,p<0.001,d=0.40) than men (Fig. 2*B*). However, men and women did not significantly differ on anhedonia scores (t(253.79)=−2.05, *p* = 0.414, *d* = −0.17; Fig. 2*C*) or levels of mean reward anticipation (t(233.53)=−2.50,p=0.131,d=−0.23; Fig. 2*D*). Levels of depressive traits (BDI), chronic stress (PSS), and reward anticipation were significantly correlated with each other; neither measure was significantly correlated with working memory ability (K estimate; Fig. 3).

### Predictors of overall high versus low effort choices

**Figure 4.**
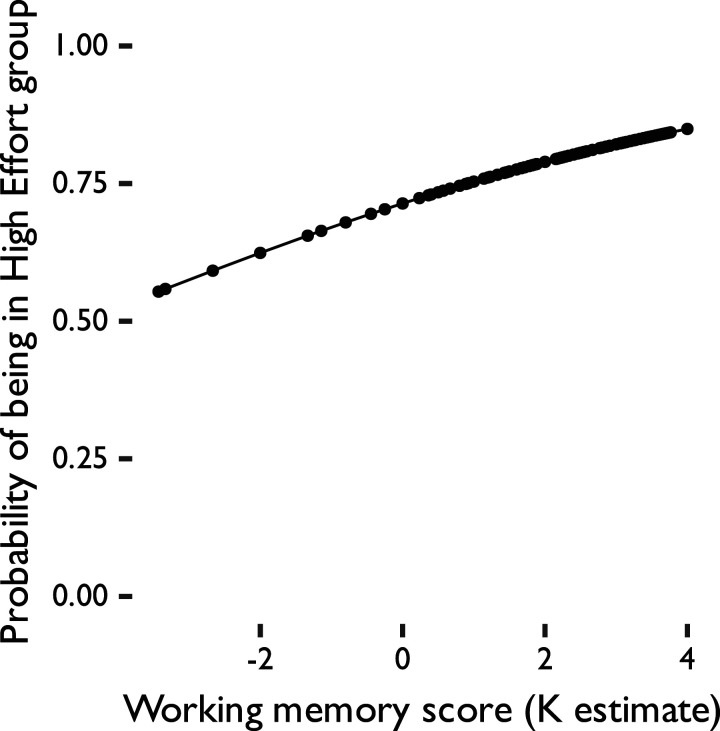
Binomial logistic regression predicting whether participants were in the high effort versus low effort group. High effort group, >70% high reward (HR) trials selected; low effort group, ≤70% HR trials selected.

Our primary research question focused on factors that predict the likelihood of choosing high versus low effort/reward options (Table 2 and Fig. 4). Here, results of the binomial regression showed that only visual short-term memory (K estimate) score significantly predicted whether a participant was in the high effort or the low effort group ($z=2.26,\;p=0.024,e^{\beta }=1.24$). Participants with higher working memory ability had a higher probability of being in the high effort group. Depressive traits, chronic stress levels, and reward anticipation did not significantly predict whether participants were in the low versus high effort groups. Furthermore, a linear model analysis with the same predictors revealed that, once again, only visual short-term memory (K estimate) scores significantly predicted the proportion of high effort trials that a participant selected (Table 3).

**Table 2.** Binomial logistic regression predicting whether participants were in the high effort versus low effort group

	Binomial logistic regression: high versus low effort group
Working memory ability (K estimate)	0.21*
	(0.09)
BDI (prop. score)	−0.78
	(0.96)
PSS	0.00
	(0.03)
Reward anticipation	0.00
	(0.07)
Num. Obs.	429
AIC	419.7
BIC	440.0
Log. Lik.	−204.833
RMSE	0.39

High effort group, >70% HR trials selected; low effort group, ≤70% HR trials selected; BDI (prop. score), depression score on the beck depression inventory; PSS, perceived stress scale. Proportion scores are scores divided by total possible score. AIC, Akaike information criterion; BIC, Bayesian information criterion; ICC, intraclass correlation; RMSE, root mean squared error.

^+^*p* < 0.1, *p < 0.05, ^**^p < 0.01, ^***^p < 0.001.

**Table 3.** Linear regression of predictors for proportion of high effort trials selected

	Linear model: proportion of high effort trials selected
(Intercept)	0.84^***^
	(0.04)
Working memory ability (K estimate)	0.02^**^
	(0.01)
BDI (prop. score)	−0.03
	(0.07)
PSS	0.00
	(0.00)
Reward anticipation	0.00
	(0.00)
Num. Obs.	429
*R* ^2^	0.018
*R*^2^ Adj.	0.009
AIC	−274.2
BIC	−249.8
Log. Lik.	143.105
RMSE	0.17

High effort group, >70% HR trials selected; low effort group, ≤70% HR trials selected; BDI (prop. score), depression score on the beck depression inventory; PSS, perceived stress scale. Proportion scores are scores divided by total possible score. AIC, Akaike information criterion; BIC, Bayesian information criterion; ICC, intraclass correlation; RMSE, root mean squared error.

^+^*p* < 0.1, *p < 0.05, **p < 0.01, ***p < 0.001.

### Accuracy

**Figure 5.**
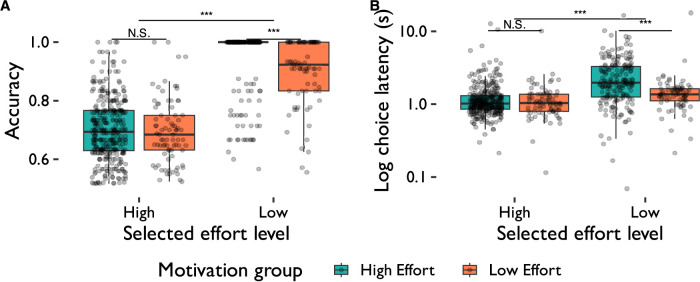
Accuracy and choice latency in the choice phase of the change detection task by motivation group (high effort vs low effort group). The choice latency plot has been log-transformed on the *y*-axis to more clearly show small choice latency values. High effort group, >70% HR trials selected; low effort group, ≤70% HR trials selected.

In all ANOVAs reported below, all *p*-values were corrected for multiple comparisons using Greenhouse–Geiser correction. A 2 × 2 within-between ANOVA (group × trial type; Table 4 and Fig. 5*A*) revealed a main effect of trial type on accuracy such that participants showed poorer performance on the more difficult HR trials compared to the easier LR trials (F(1,273)=724.10,p<0.001,γ=0.515). This was qualified by a trial type × group interaction such that participants in the high effort group who selected high effort trials more than 70% of the time performed better than those in the low effort group on LR but not HR trials (F(1,273)=11.81,p<0.001,γ=0.017).

**Table 4.** Summary table of accuracy and choice latency by group and effort level chosen

Group	Effort level	*M*_Accuracy_	SD_Accuracy_	*M*_Latency_	SD_Latency_
High effort	High	0.70	0.11	1.27	1.04
High effort	Low	0.95	0.11	2.54	1.99
Low effort	High	0.70	0.11	1.23	1.10
Low effort	Low	0.90	0.11	1.60	1.92

High effort group, >70% HR trials selected; low effort group, ≤70% HR trials selected.

### Choice latency

A 2 × 2 within-between ANOVA (group × trial type; Table 4 and Fig. 5*B*) revealed a main effect of trial type such that participants were slower to choose HR trials compared to LR trials (F(1,273)=37.50,p<0.001,γ=0.063). Furthermore, high effort participants were slower than low effort participants to make choices across all trial types (F(1,273)=10.10,p=0.002,γ=0.019). There was also a trial type × group interaction, such that participants in the high effort group took longer to select LR trials than HR trials, while choice latency between high effort and low effort groups did not differ on HR trials (F(1,273)=37.50,p<0.001,γ=0.021).

### Additional predictors of accuracy and choice latency

**Figure 6.**
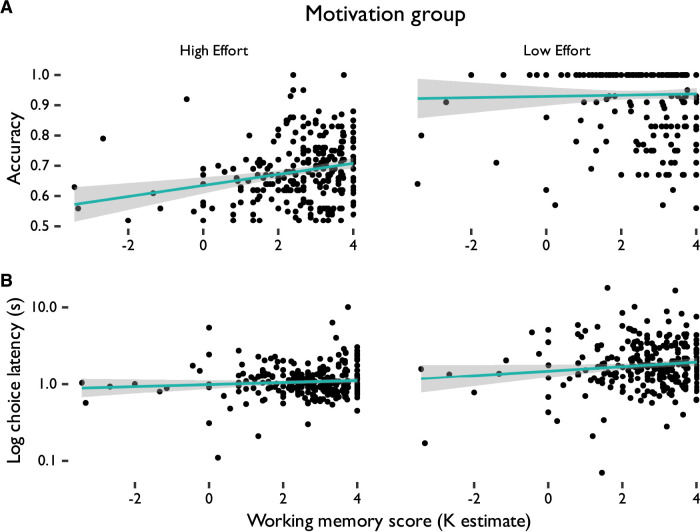
***A***, Accuracy and ***B***, choice latency in the reward phase of the change detection task by working memory ability (K estimate). The choice latency plot has been log-transformed on the *y*-axis to more clearly show small choice latency values.

We additionally evaluated predictors of accuracy on each trial and choice latency for selecting the trial difficulty from individual differences in participant visuospatial short-term memory capacity, mood disorder score, and reward anticipation by sex. A multi-level model analysis revealed that the effort level required in the selected trial significantly predicted accuracy across all trials such that accuracy was higher on LR trials compared to HR trials. Importantly, of the predictors of interest, only visuospatial short-term memory score predicted accuracy, such that higher short-term memory ability was associated with higher accuracy (Table 5 and Fig. 6*A*). Trial effort level also predicted choice latency, with slower choices on low relative to high effort trials. Depression scores also predicted choice latency such that higher depression scores predicted faster choices across all trials (Table 6 and Fig. 6*B*). Sex, chronic stress scores, and reward anticipation did not significantly predict accuracy or choice latency.

**Table 5.** Multi-level model analysis coefficients and standard errors for accuracy in the reward phase of the task

	Multi-level model: accuracy
(Intercept)	0.64^***^
	(0.02)
Sex	0.01
	(0.01)
Working memory ability (K estimate)	0.01^**^
	(0.00)
BDI (prop. score)	−0.01
	(0.04)
PSS	0.00
	(0.00)
Reward anticipation	0.00
	(0.00)
Effort level	0.25^***^
	(0.01)
SD (intercept participant)	0.04
SD (observations)	0.10
Num. Obs.	542
*R*^2^ Marg.	0.592
*R*^2^ Cond.	0.656
AIC	−840.0
BIC	−801.3
ICC	0.2
RMSE	0.09

BDI (prop. score), depression score on the beck depression inventory; PSS, perceived stress scale. Proportion scores are scores divided by total possible score. AIC, Akaike information criterion; BIC, Bayesian information criterion; ICC, intraclass correlation; RMSE, root mean squared error.

^+^*p* < 0.1, *p < 0.05, **p < 0.01, ***p < 0.001.

**Table 6.** Multi-level model analysis coefficients and standard errors for choice latency in the reward phase of the task

	Multi-level model: choice latency
(Intercept)	1.25^***^
	(0.34)
Sex	−0.07
	(0.17)
Working memory ability (K estimate)	0.05
	(0.05)
BDI (prop. score)	−1.11*
	(0.54)
PSS	0.01
	(0.01)
Reward anticipation	−0.04
	(0.04)
Effort level	1.06^***^
	(0.13)
SD (intercept participant)	0.18
SD (observations)	1.53
Num. Obs.	542
*R*^2^ Marg.	0.114
*R*^2^ Cond.	0.127
AIC	2042.9
BIC	2081.6
ICC	0.0
RMSE	1.51

BDI (prop. score), depression score on the beck depression inventory; PSS, perceived stress scale. Proportion scores are scores divided by total possible score. AIC, Akaike information criterion; BIC, Bayesian information criterion; ICC, intraclass correlation; RMSE, root mean squared error.

^+^*p* < 0.1, *p < 0.05, **p < 0.01, ***p < 0.001.

### Drift diffusion model

**Figure 7.**
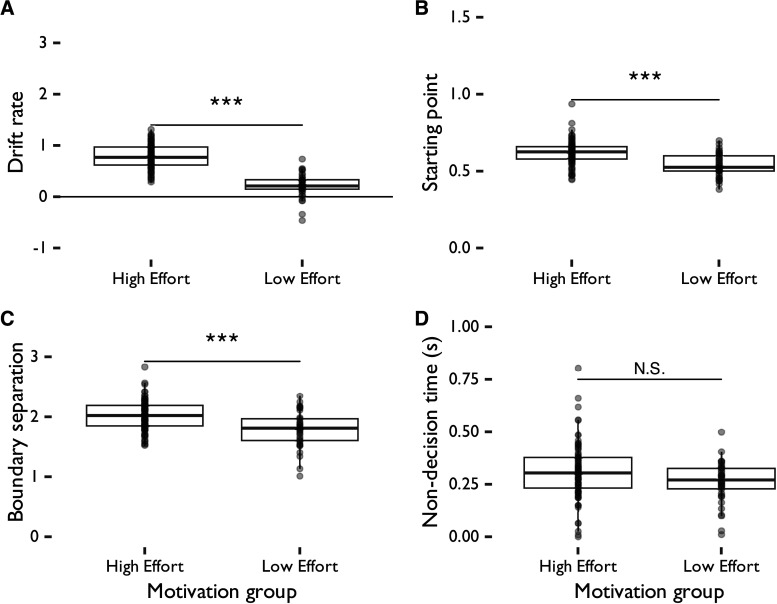
Outputs of a hierarchical Bayesian drift diffusion model fit to all participants in the effort choice task, divided by effort group. High effort participants had a significantly higher ***A***, drift rate, ***B***, starting point and ***C***, boundary separation compared to low effort participants, but did not significantly differ on ***D***, non-decision time compared to low effort participants.

To evaluate group differences in evidence accumulation and bias toward deploying higher amounts of cognitive effort in decision-making when choosing HR or LR trials, we fit a hierarchical Bayesian drift diffusion model (Ahn et al., 2017) to choice (HR vs LR trial selected) and corresponding choice latency data on each trial from all participants who had at least one correct response on an HR and an LR trial and who chose at least four trials of each type (N=136, *n*_High effort_ = 94, *n*_Low effort_ = 42). A drift diffusion model can only be fit to participants who selected both trial choices. For this reason, we excluded data from *n* = 2 participants who selected only low effort trials and *n* = 86 participants who selected only high effort trials. We then evaluated this overall model fitting data from these participants to explore group-level differences in whether participants selected HR or LR trials more often. For each participant in the high effort and low effort groups, we evaluated four parameters: drift rate (speed of evidence accumulation in deciding on an HR vs LR trial), starting point (bias toward HR vs LR trial), boundary separation (extent to which speed and effort choice are traded off between HR and LR trials), and non-decision time (choice latency time unrelated to trial choice, such as time for motor response; Fig. 7). These parameters capture individual differences in the speed and motivation to make a decision about deploying more or less cognitive effort for reward. After Bonferroni correction for multiple comparisons, we found that high effort group participants had a starting point closer to the HR trial decision boundary (t(83.21)=5.80,p<0.001,d=1.05); had wider decision boundaries (t(68.49)=4.38,p<0.001,d=0.87) than low effort group participants; and had a significantly higher drift rate (t(82.94)=13.54,p<0.001,d=2.46) than low effort group participants. However, participants did not significantly differ in non-decision time (t(105.65)=2.34,p=0.213,d=0.39). These findings suggest that participants who were in the high effort group required more evidence to select low but not high effort trials, were more strongly biased toward selecting high effort/HR trials, and were quicker to accumulate evidence for these choices, than those in the low effort group.

To evaluate the quality of our DDM analysis, we conducted a parameter recoverability test by fitting this DDM to simulated binary choice and response time data that we generated using a Wiener process in the rwiener R package (Wabersich and Vandekerckhove, 2014) with the same average drift rate, starting point, boundary separation, non-decision time, and number of samples as our actual data. We evaluated the goodness of fit of the model to the simulated and the actual data using the leave-one-out cross validation information criterion (LOOIC) measure. Our model showed better parameter recoverability to the simulated data (LOOIC = −8,997.31) compared to the actual data (LOOIC = 8,080.26). However, the parameter fits were significantly correlated between simulated and actual data (t(134)=2.94,p=0.039,r=0.25), suggesting good parameter recoverability in our model.

## Discussion

In this study, we investigated whether working memory performance, depressive traits, and chronic stress levels influence people’s motivation and ability to consistently deploy cognitive effort for reward. Results showed that greater working memory ability significantly predicted the likelihood of systematically choosing high effort/HR trials (whether the participant was classified in the high vs low effort group), whereas chronic stress, depression trait levels, and reward anticipation were not predictive of effort deployment choices. Additionally, participants required more evidence to select LR trials compared to HR trials. DDM further indicated that those who were categorized as high effort participants required less evidence to select high effort/HR trials, and were more strongly biased toward selecting these trials, than low effort participants. Furthermore, working memory ability significantly predicted accuracy, while depressive traits predicted choice latency; both factors significantly differed depending on the effort required in the trial.

These results partially recapitulate findings from rodent research using the rCET regarding differences in performance and decision-making biases between those who select high effort trials more often and those who select them less often. As by Cocker et al. (2012), participants who chose high effort trials over 70% of the time performed better than those who chose high effort trials 70% or less of the time, on LR—although not HR—trials. Continually deploying high amounts of cognitive effort could be fatiguing; the high effort group’s lower performance on HR trials could reflect the ineffectiveness of this high effort choice strategy, or the fact that those with higher visuospatial short-term memory ability (*K* > 3) had a more difficult task with higher effort demands on high effort trials.

High effort group participants, who generally selected HRs more often and who were more biased toward selecting HR trials than low effort group participants, appear to have spent more time deliberating before selecting an LR trial than low effort group participants. Choice latency indicates how long participants spend when deciding which effort level to choose. Thus, they may have been trading off the benefits and costs of selecting a LR trial more consciously than low effort group participants were (Sandra and Otto, 2018; Pruessner et al., 2020), even as they performed significantly better than low effort group participants on LR but not HR trials. These tradeoffs could shift over time in the task as participants become fatigued or bored with the task, or found a new effort deployment strategy that better optimized rewards or task enjoyment.

Furthermore, our findings align with past work in rodents (Hales et al., 2024) in that our drift diffusion model results showed wider decision boundaries between HR and LR trials for high effort group compared to low effort group participants, as well as a starting point closer to HR trials for high effort group participants. Thus, we are the first to show in humans that evidence accumulation for low effort short-term memory tasks can be greater if participants consistently deploy high levels of effort. Given that the decision available on each trial is the same, individual differences in this initial level of bias toward selecting high effort trials—together with random noise—drive differences in trial difficulty choice between participants as modeled by the drift diffusion model. Therefore, the drift diffusion model may not be capturing meaningful, systematic patterns of effort deployment choices. However, it still provides an exploratory evaluation of the underlying cognitive processes that could be driving effort deployment choices. Additionally, as with rodents, participants in the low effort group made faster choices than those in the high effort group on LR trials. Furthermore, high effort group participants showed steeper drift rates than low effort group participants—while rodents in the equivalent high effort group had steeper drift rates when they subsequently made correct responses for their given choice (Hales et al., 2024). Taken together, these findings could suggest similarities between humans and rodents in the influence of cognitive capabilities on performance when deploying more cognitive effort, as well as similarities in biases toward deploying more effort or wanting higher rewards. Furthermore, given the conditionality of the drift rate findings in rodents, strategies driving impulsiveness or choice motivations to select an HR or LR trial could differ between humans and rodents. This could be explained by differences in the strength of reinforcement provided by the reward, which was a secondary reinforcement (points toward a monetary reward) in our task but a primary reinforcement (sugar pellets) in the rCET (Cocker et al., 2012; Hales et al., 2024), or by other differences between our task and the rCET as well as between humans and rodents in terms of how reward motivation is processed. These differences were not driven by reward anticipation, which did not differ between high and low effort groups (t(122.95)=0.17,p=1,d=0.02).

Additionally, previous studies with the rCET have shown no overall accuracy differences between high and low effort group participants across either LR or HR trials (Hosking et al., 2016; Silveira et al., 2020). However, in a recent, larger analysis, Hales et al. (2024) showed that rodents choosing more HR trials were slightly more accurate on HR than LR trials when completing the trials themselves, while rodents choosing more LR trials were slightly more accurate on LR than HR trials when completing these trials. Although participants practiced task contingencies ahead of the main choice phase and the task difficulty was calibrated to working memory ability, individual differences in working memory—which predicted whether a participant selected high or low effort trials more often—still predicted these performance differences in humans. Furthermore, chronic stress—which can negatively impact availability of executive resources—did not explain accuracy or choice latency in this task. Thus, within the constraints of a binary effort choice task, participants’ willingness to deploy cognitive effort was explained more by individual differences in executive function (working memory capability; Sandra and Otto, 2018) rather than affective factors or the effects of affective factors on executive function (Slattery and Cryan, 2017). Note that although we did not measure executive control as a global construct in this study, visuospatial working memory ability is typically described as a process that falls withing the suite of executive functions (Miyake and Friedman, 2012).

Our drift diffusion model results also suggest that high effort group participants had to overcome a bias toward HR trials in order to select LR trials. This process could be driven by adjustments to effort deployment strategies based on performance, as suggested by recent interpretations of the expected value of control theory (Shenhav et al., 2021). Through this framework, choosing a high proportion of low effort trials could be seen as optimizing effort deployment and making slower and more considered decisions based on performance—as reflected in increased choice latency among high effort group participants for LR trials—whereas selecting a high proportion of high effort trials could entail more rapid and impulsive decisions. In order to evaluate whether participant performance was driven by trial-to-trial, model-free strategies—as opposed to a more considered strategy that incorporates performance over multiple trials—we conducted a follow-up linear regression analysis to evaluate whether performance on the previous trial predicts performance on the current trial as a function of effort motivation group and trial type. Performance on the prior trial, but not effort motivation group, significantly predicted performance on the current trial (t(68955)=8.45,p<0.001). This suggests that, overall, participants maintained a constant performance-based strategy that was not explained by effort choice decisions. Furthermore, participants with higher levels of depressive traits responded significantly faster across all trials, a result that runs counter to past work suggesting increased choice latency in reward tasks in rodents (Hales et al., 2023) or humans (Di Schiena et al., 2013) with depressive-like traits. However, after Bonferroni correction, depressive traits and choice latency were not significantly correlated across all participants and trials (t(702)=−1.21,p=1,r=−0.05). As depressive traits did not impact accuracy, these findings could instead suggest a lack of engagement with decision-making in the task characterized by higher levels of automatic responding given high levels of depressive traits (Teachman et al., 2012).

Our findings suggest that choices to deploy cognitive effort for reward are primarily driven by participants’ cognitive capacity (as measured by visuospatial working memory ability) and not by depressive traits, chronic stress, or reward anticipation, in participants within a non-clinical range of depression traits and chronic stress. Potentially, the aversiveness of expending cognitive effort for participants with lower working memory capacity may have overridden overall sensitivity for reward. Alternatively, participants with lower working memory abilities may have determined that they were unlikely to do well on HR trials, and so may have been less incentivized to deploy effort on HR trials. Additionally, although our population exhibited a wide range of depression scores—including those above clinical thresholds (Wang and Gorenstein, 2013)—this population was non-clinical. In contrast, much work on the impact of reward anticipation on cognitive flexibility and related constructs has focused on participants who were clinically diagnosed with depression (Alloy et al., 2016; Terpstra et al., 2023) or on rodents in which chronic stress was directly induced (Watt et al., 2017). In order to obtain the highest accuracy and reward possible, participants may have titrated the task’s difficulty—through choosing a higher proportion of LR trials—to a level at which they can consistently complete the task and at which the value of their effort was greatest (Shenhav et al., 2013).

We found no significant sex differences in performance or choice latency in our study. Women generally present more with depression than men (Parker and Brotchie, 2010) and are also more impacted by chronic stress across the lifespan (Hodes and Epperson, 2019). However, the results of our study were primarily driven by visuospatial short-term memory ability, where sex differences are overall smaller and women may be better than men at memory for location (Voyer et al., 2017)—a relevant measure in our task. Our undergraduate psychology student sample strongly skewed female; in future studies, it would be important to obtain a more balanced sample in order to further explore potential sex differences.

There are a number of caveats we must consider when interpreting these results. First, the study was conducted fully online on participants’ own devices. While this allowed us to obtain a larger and better powered sample, differences in screen size and background distractions could have impacted participants’ ability to encode the positions and colors shapes on each trial. These differences may have made the task more difficult than originally intended for some participants. However, participant performance was still near ceiling on LR trials and very high on HR trials, so this was unlikely to have a large effect.

Second, high effort trials never gave LRs, or vice versa. This may impact our ability to evaluate whether it was sensitivity to effort or to reward that more strongly drove participant choices in the task. However, reward anticipation did not predict choices for high effort deployment. This suggests that effort costs could be weighed more strongly than rewards by participants when making effort choices. In future studies, varying levels of effort and reward independently would allow us to explore whether differences in reward anticipation for small versus large rewards differentially influence decisions to deploy effort for reward. For example, if a participant only chooses to deploy effort for large rewards as opposed to for all rewards, they may pursue a strategy of only selecting trials that give large rewards regardless of the required effort level. This would allow us to better understand the role of reward magnitude in motivating effort choices.

Third, the set sizes used on the change detection task were modified from those originally used by Luck and Vogel (1997). The set size of 10 was removed from the pool of trials as pilot participants had very low performance at this set size. Although this change reduced the possible variety of trials used, participants still reported a maximum set size of eight to be highly challenging. Furthermore, the choice structure of the task incorporated only two of the set sizes. This could have made the task excessively easy for some participants—as reflected in the ceiling effect on LR trials—and excessively difficult to others. However, we did titrate task difficulty according to participants’ visual short-term memory ability, reducing the likelihood of the task difficulty not matching participant ability. Future studies could use a finer-grained, parametric design that offers choices at more difficulty levels to examine whether motivation continuously varies with effort levels (Sayalı et al., 2023).

Fourth, the method used to calculate the K estimate slightly differed between the task—used to set the criterion level—and our analysis—used to establish the K estimate for each participant. In the task, the K estimate was calculated based on the current hit and false alarm rate after every trial, with the final K estimate being used to set the criterion for the choice phase of the task. In our analysis, we calculated the K estimate based on the full hit and false alarm rate at each set size. Because of rounding differences in these calculations, the K estimate values used in our analysis differ from those in our task by M=0.01,SD=0.63. We consider this difference to be small enough as to not have an effect on the difficulty of the task.

Fifth, participants were told that the points they received would be proportional to the monetary reward received, which could motivate them toward higher effort choices regardless of their effort capabilities—shifting their effort/reward tradeoffs. However, such a strong effort incentive was necessary to motivate participant performance given the difficulty of the high effort trials for many participants. Participants may have also individually differed in compliance with reading instructions or in their motivation for reward. Such factors could be investigated in a follow-up study incorporating qualitative evaluations of participant experiences of the task.

Sixth, differences in performance that are explained by the K estimate could also be explained by the differences in set sizes available associated with being above or below the K criterion of 3 on set size = 4. However, the set sizes at each level of criterion were established with piloting such that the difficulty of each criterion level was matched as closely as possible. Last, although our task only had a single probe whose color had to be compared to the original probe, participants had to attend to the whole display to compare the single probe to the existing square at any given location onscreen. As such, we used the whole-display K estimate measure to calibrate participant performance in our task (Rouder et al., 2011). A single-probe K estimate measure reflects lower effort demands than the whole-display measure, as the whole-display measure requires the status of all shapes onscreen to be held in memory for the probe—whereas the single-probe measure only involves evaluating one shape in one position. However, as the position of the probe shape in our task is randomized, participants must still attend to the whole display to correctly identify any changes in the probe’s color. As such, the whole-display K estimate measure is more relevant for our task’s structure and cognitive effort demands, and the results could differ if we use the single-probe measure.

Future studies could make further use of information about effort deployment choices that can only be captured in human studies. As many human cognitive effort tasks—unlike rodent tasks—do not require substantial training, a future study could examine effort choices given a wider range of effort and reward levels, as well as test how preference for effort changes given high effort trials with LR and vice versa. Such a task could more richly capture individual differences in human cognitive effort deployment decisions. Furthermore, an expanding field of research uses qualitative approaches to investigate why participants make the judgments they do about effort deployment, as well as ask about participant experiences of task difficulty and the value of rewards versus the effort required to obtain them (Vásquez-Rosati et al., 2019). Understanding the phenomenology of cognitive effort deployment choices would help us extend our understanding of how and why we make decisions to deploy more or less cognitive effort for reward, beyond traditional self-report measures. Participant descriptions of their experiences in the task could indicate whether boredom and fatigue, in addition to reward motivation, impacted effort deployment choices. This approach would also allow us to further explore how rodent-based constructs of cognitive effort deployment are reflected in human decision-making, while extending our understanding of these constructs with human-specific experiential information. Additionally, exploring how the brain represents information about effort task difficulty—especially in regions relevant to cognitive control like the dorsal anterior cingulate cortex (Shenhav et al., 2016; Yee et al., 2021)—through functional magnetic resonance imaging would help us better understand how motivational states in cognitive effort decision-making are reflected in neural circuitry.

Our study suggests that, as in rodents, significant individual differences exist in the human tendency to choose harder but more rewarding options in cognitive effort tasks. Using a visuospatial working memory task, we show that individual differences in accuracy on the trial and choice latency for the trial type chosen are primarily driven not by depressive traits or chronic stress—as has previously been shown in the rodent literature—but instead by working memory capability. These findings may help to inform clinical interventions aimed at increasing motivation to seek rewards and engage in work in everyday life, and illustrate the importance of translating and extending rodent work with human-specific measures.

## Data and Code Availability

All task code, stimuli, data, and code used to generate this manuscript and the figures are available at https://osf.io/c4h7s/.

## Synthesis

Reviewing Editor: Ifat Levy, Yale School of Medicine Decisions are customarily a result of the Reviewing Editor and the peer reviewers coming together and discussing their recommendations until a consensus is reached. When revisions are invited, a fact-based synthesis statement explaining their decision and outlining what is needed to prepare a revision will be listed below. The following reviewer(s) agreed to reveal their identity: Ceyda Sayali, Douglas Lee.

This study explores how visual short-term memory capacity, chronic stress, depressive traits, and reward sensitivity influence decision to exert cognitive effort. Utilizing an online visual short-term memory task in undergraduate participants, the research distinguishes between high effort and low effort groups based on their choices to engage in tasks requiring varying levels of cognitive effort for rewards. The results reveal that greater working memory ability is a significant predictor of a participant's likelihood to choose high-effort/high-reward tasks, while chronic stress, depressive traits, and reward sensitivity do not predict effort deployment choices. The paper also demonstrates some similarities between rat and human behavior with respect to cognitive effort allocation.

The paper addresses an important research question, and is well-written and easy to follow. The motivation and background, as well as the experimental design and analysis, are clearly explained. The adaptation of a task from rodent models offers valuable insights into the complex interplay between cognitive functions and motivational states across species. Reviewers identified, however, certain limitations that should be addressed, as detailed below.

Introduction:

- pg 5. Par 2. The paragraph introduces several important concepts - differences between humans and rodents in studying cognitive effort, the role of executive functions, the importance of visuospatial working memory, and how reward sensitivity affects neural circuitry - without a clear statement that ties these ideas together into a cohesive argument or narrative. This leads to a sense of fragmentation where the reader is presented with interesting statements but no clear understanding of how they collectively advance the argument or understanding of the topic. While the paragraph provides a rich overview of relevant literature and concepts, it could be improved by focusing on a clearer central message, reducing information overload, providing more context for the introduced concepts, and aligning more closely with the study's main objectives or hypotheses.

- Pg 6 Par 1: "However, the degree to which individual differences in reward sensitivity and executive function capacity influence the likelihood of choosing to deploy more cognitive effort for higher reward is not known." This statement is inaccurate: both reward sensitivity and executive function capacity have been correlated with effort discounting. For example, studies have shown that dopamine plays a significant role in evaluating the costs and benefits of expending effort for rewards, affecting individuals' decisions to choose more effortful options when higher rewards are anticipated. Just to name a few references:

Michely, J., Viswanathan, S., Hauser, T. U., Delker, L., Dolan, R. J., &Grefkes, C. (2020). The role of dopamine in dynamic effort-reward integration. Neuropsychopharmacology, 45(9), 1448-1453. Westbrook, A., Kester, D., &Braver, T. S. (2013). What is the subjective cost of cognitive effort? Load, trait, and aging effects revealed by economic preference. PloS one, 8(7), e68210.

Westbrook, A., Van Den Bosch, R., Määttä, J. I., Hofmans, L., Papadopetraki, D., Cools, R., &Frank, M. J. (2020). Dopamine promotes cognitive effort by biasing the benefits versus costs of cognitive work. Science, 367(6484), 1362-1366.

Raghunath, N., Fournier, L. R., &Kogan, C. (2021). Precrastination and individual differences in working memory capacity. Psychological Research, 85(5), 1970-1985.

Methods:

- The decision to utilize the rodent Cognitive Effort Task (rCET) without referencing or comparing it to other established measures like the Cognitive Effort Discounting Paradigm (COGED) represents a missed opportunity in the study's methodological rigor. This omission limits readers' understanding of the task's relative merits and suitability for investigating the research question. Providing a more thorough comparison or justification for the selection of the rCET could have enhanced the transparency and robustness of the study's methodology.

- Moreover, the design choice of the study, contrasting only two levels of cognitive effort (easier vs. harder tasks) with directly associated rewards (low vs. high), has limitations compared to the parametric design employed in Cog-ED. First, a parametric design, offering multiple levels of effort and reward, would allow for a more nuanced understanding of how different degrees of effort influence decision-making. It can uncover dose-dependent relationships, providing richer insights into the dynamics between effort and reward.

Second, by always pairing high effort with high reward, and vice versa, the study's design conflates the effects of effort and reward sensitivity, making it difficult to disentangle which factor - effort or reward - more strongly influences a participant's choice. This limitation restricts the ability to fully understand the differential contributions of reward versus effort sensitivity in cognitive effort deployment.

- Why was the potential reward for high vs. low effort trials 10x greater even though the effort level was probably nowhere near 10x greater? Was it to greatly exaggerate the benefit-cost ratio for high effort trials relative to low effort trials, so that more people would choose high effort more often?

- Pg 11 line 224-225: Why was the N=8 condition not practiced although presented later? Is this a typo or a design choice? Please explain.

- Lines 239-243: Would any results differ if the single-probe K estimate measure had been used instead of the whole-display K estimate measure?

- Line 270 (and elsewhere): Why was the K estimate measure calculated based on the set size of 4?

- Pg 13. "Although participants were told that the number of points they gained in this phase was proportional to the monetary reward they would receive, all participants received the same reward"

Instructing participants that the points gained would be proportional to the monetary reward, while ultimately providing the same reward to all, can introduce a bias towards selecting the harder task. This setup potentially confounds the study's aim to objectively measure cognitive effort deployment, as participants might be overly motivated by the prospect of a higher payout, skewing results towards high-effort choices irrespective of their natural inclination or capacity for effort. Such a design might not accurately reflect true effort-reward valuation, compromising the study's ability to distinguish between the intrinsic motivations for choosing different levels of cognitive effort. Moreover, individual differences in complying with instructions, or reward motivation (independent of effort) may also influence the findings. The authors are encouraged to visit the literature on human cognitive effort avoidance to review the format of their instructions and discuss how their instructions deviated from this literature.

Results:

- Pg 14. By classifying participants into just two categories based on a 70% cutoff for choosing the high-reward (HR) option, the study reduces the variability of participants' choices to a binary distinction, which may oversimplify the complex nature of cognitive effort deployment. This categorization overlooks the nuances and variability in individual decision-making processes, potentially masking subtler trends and relationships within the data. Analyzing choices as a continuous variable could provide a more detailed and nuanced understanding of how various factors influence the propensity to exert cognitive effort, offering richer insights into individual differences in effort-reward valuation.

- Lines 352-353: The fact that participants with higher working memory scores had a higher probability of being in the high effort group might simply be due to authors using K=3 as a binary cutoff to determine the effort levels that would be available for each participant to choose from in the choice task. But, some people might have been K=8 if the authors had allowed for that, which means that they would always choose high effort because it was actually easy for them than it would be for people with lower K scores. For example, maybe those participants who should have been classified as K=8 would have chosen fewer high effort trials if they were offered the choice between 6 boxes and 10 boxes.

- How correlated are the measures for depression, stress, reward anticipation, and working memory?

- Pg 18. Please report the actual values for accuracy / descriptive stats in a table or in text.

- DDM Modeling: What is the evidence that is accumulating during the decision? DDM analysis usually relies on the theoretical foundation that there is clear evidence that is accumulating (e.g., physical motion energy for perceptual tasks or value estimation for preferential tasks). Here, it is not clear what the evidence would be. The author should try to offer a theoretical motivation for why a DDM should explain this sort of decision.

- Line 391: Is it sufficient to have only one trial of either LR or HR when fitting the DDM for a participant?

- General: Since all trials offer the same decision, why wouldn't a participant simply make the same choice on every trial? In other words, under the DDM framework, are all choices based purely on the diffusion noise combined with the starting point bias?

- Overall results/discussion: The study's reliance on a binary task difficulty model (easy vs. hard) potentially obscures nuanced individual responses to cognitive effort. High working memory (WM) individuals performing almost at 100% on easier tasks suggests a mismatch between task demand and participant capability, possibly reflecting avoidance of boredom rather than effort. This is evidenced by High Effort participants' significant performance advantage in LR but not HR trials compared to Low Effort group and their initial bias against the easy task, which persists throughout the study. A parametric design, offering a gradient of difficulty levels, could better match tasks to individual abilities, avoiding ceiling effects and more accurately capturing the dynamics of motivation, effort, and enjoyment (Csikszentmihalyi's concept of flow, also covered in effort avoidance design in Sayalı, C., Heling, E., &Cools, R. (2023)). This approach would allow for a finer understanding of how cognitive capacity influences task preference beyond simplistic effort avoidance, emphasizing the importance of task engagement and its relation to cognitive abilities.

Sayalı, C., Heling, E., &Cools, R. (2023). Learning progress mediates the link between cognitive effort and task engagement. Cognition, 236, 105418.

- Discussion: The authors could revise the paper to more critically discuss the limitations of their current study design and interpretation of results. This includes acknowledging the potential for ceiling effects in easier tasks and the implications of using a binary classification system for effort choices. They could also explore the nuances of their findings in the context of existing human literature on effort and reward, including discussions on the potential for boredom or task engagement influencing choices, perhaps as a distinct factor that differentiates humans from rats. Future research directions could be proposed, highlighting the value of parametric designs and continuous measures for capturing a broader spectrum of cognitive effort deployment.

Minor comments

- Figure 1 legend: Instead of "2 or 4, or 6 or 8" it should read "2 or 6, or 4 or 8" according to what is described in the text.

- Line 272 (and elsewhere): It seems that the authors sometimes refer to "reward sensitivity" although at other times they refer to it as "reward anticipation". Are these the same thing? If so, I recommend using "anticipation" in all instances. "Sensitivity" gives the impression that they are better or worse at distinguishing between reward levels, although the authors clearly describe that they are interested in how motivating rewards are in general to an individual participant.

- Line 289: Perhaps "motivation" should be changed to something like "tendency".

- Line 317 (and elsewhere): Although the authors make it clear that the RT they examine is for the decision about which task to perform and not for the task itself, it may sometimes be confusing for the reader. This is because the authors mention RT in the same sentences or paragraphs as they mention accuracy, which does refer to the performance within the task itself. Perhaps they can find a way to rephrase the relevant sections.

- Line 366: "Group" should perhaps be replaced by "Trial Type".

- Line 398: Perhaps remove the word "portion".

- Line 407: The way it's phrased, this is not quite accurate. High effort participants needed more evidence before selecting the low effort trials, but not necessarily the high effort trials (because of the starting point bias).

- Lines 413-415: Doesn't this just show that working memory ability is continuous rather than binary?

- Lines 417-419: Perhaps add that they required more evidence for low effort/reward trials.

- Figure 6: Please explain if boundary separation is from 0 to X or from -X to X. Then, please also explain what a starting point of 0.5 means with respect to X. For example, if boundary for a participant = 1.5, is the lower boundary 0 or -1.5... and is the starting point a percentage of the different between the boundaries or what?

- Line 429: Perhaps "less" should read "more"?

- Lines 438-439: Did reward sensitivity differ across groups?

- Line 439: The authors contrast their results against results from a previous study where there were correct and incorrect answers, but it's unclear how the concept of "correct" would apply in the current study.

- Lines 443-445: This statement is not clear.

- Lines 454-457: The way it is phrased, it is not clear what the comparison is between when stating that certain rodents were "more accurate".

- Lines 489-490: But why would this vary across trials?

- Lines 497-501: It seems that such an explanation would require the authors to include in their analysis a measure of performance on trials prior to the current trial.

- This paragraph was hard to follow. Furthermore, it seems that this sort of explanation should be mentioned earlier.

- There are several very minor typos in the manuscript, so a solid proofing for such minor typos would be advised.

## Author Response

We would like to thank the reviewers for their overall positive responses and highly constructive comments, and we are pleased to have the opportunity to revise and resubmit the paper. We feel it is much stronger as a result of their feedback.

In response to reviewer comments, we made several important changes to the manuscript. In addition to revising and clarifying sections of the text in response to reviewer points, we have run additional linear regressions evaluating whether performance on the previous trial predicts performance on the current trial as well as whether the proportion of high effort trials chosen was predicted by short term memory ability, depressive traits, chronic stress, and reward anticipation. Please also note that, due to TeX conversion inconsistencies between our computer and the submission system, the titles of referenced papers may be partially cut off in the PDF document; we apologize for any inconvenience this may cause.

We address the reviewer comments point by point below.

This study explores how visual short-term memory capacity, chronic stress, depressive traits, and reward sensitivity influence decision to exert cognitive effort. Utilizing an online visual short-term memory task in undergraduate participants, the research distinguishes between high effort and low effort groups based on their choices to engage in tasks requiring varying levels of cognitive effort for rewards. The results reveal that greater working memory ability is a significant predictor of a participant's likelihood to choose high-effort/high-reward tasks, while chronic stress, depressive traits, and reward sensitivity do not predict effort deployment choices. The paper also demonstrates some similarities between rat and human behavior with respect to cognitive effort allocation.

The paper addresses an important research question, and is well-written and easy to follow. The motivation and background, as well as the experimental design and analysis, are clearly explained. The adaptation of a task from rodent models offers valuable insights into the complex interplay between cognitive functions and motivational states across species. Reviewers identified, however, certain limitations that should be addressed, as detailed below.

We thank the reviewers for their kind words and their thorough and thoughtful responses, which we have addressed below (author responses are indicated with "Response:"; revisions in text are in bold).

Introduction:

- pg 5. Par 2. The paragraph introduces several important concepts - differences between humans and rodents in studying cognitive effort, the role of executive functions, the importance of visuospatial working memory, and how reward sensitivity affects neural circuitry - without a clear statement that ties these ideas together into a cohesive argument or narrative. This leads to a sense of fragmentation where the reader is presented with interesting statements but no clear understanding of how they collectively advance the argument or understanding of the topic. While the paragraph provides a rich overview of relevant literature and concepts, it could be improved by focusing on a clearer central message, reducing information overload, providing more context for the introduced concepts, and aligning more closely with the study's main objectives or hypotheses.

Response:

We thank the reviewer for their comments and suggestions. To improve clarity and better contextualize the ideas introduced, we have accordingly rephrased the paragraph on pages 5-6 to introduce the role of attentional control in cognitive effort, a discussion of how impairments to reward anticipation and executive function capacity could drive maladaptive effort deployment, and how the impacts of these factors on decisions to deploy cognitive effort are easier to characterize in humans than in rodents - while emphasizing the importance of translating rodent work into human tasks and accounting for differences in human and rodent experiences of effort deployment and reward.

- Pg 6 Par 1: "However, the degree to which individual differences in reward sensitivity and executive function capacity influence the likelihood of choosing to deploy more cognitive effort for higher reward is not known." This statement is inaccurate: both reward sensitivity and executive function capacity have been correlated with effort discounting. For example, studies have shown that dopamine plays a significant role in evaluating the costs and benefits of expending effort for rewards, affecting individuals' decisions to choose more effortful options when higher rewards are anticipated. Just to name a few references:

Michely, J., Viswanathan, S., Hauser, T. U., Delker, L., Dolan, R. J., &Grefkes, C. (2020). The role of dopamine in dynamic effort-reward integration. Neuropsychopharmacology, 45(9), 1448-1453.

Westbrook, A., Kester, D., &Braver, T. S. (2013). What is the subjective cost of cognitive effort? Load, trait, and aging effects revealed by economic preference. PloS one, 8(7), e68210.

Westbrook, A., Van Den Bosch, R., Määttä, J. I., Hofmans, L., Papadopetraki, D., Cools, R., &Frank, M. J. (2020). Dopamine promotes cognitive effort by biasing the benefits versus costs of cognitive work. Science, 367(6484), 1362-1366.

Raghunath, N., Fournier, L. R., &Kogan, C. (2021). Precrastination and individual differences in working memory capacity. Psychological Research, 85(5), 1970-1985.

Response:

We thank the reviewer for feedback, and have revised this section to discuss past work on individual differences in cognitive effort deployment choices related to DA and 5HT processes (page 6): "Past work suggests that dopaminergic processes motivate physical and cognitive effort deployment decisions to maximize rewards (Michely, Viswanathan, Hauser, Delker, Dolan, and Grefkes, 2020), and to weigh rewards more heavily than effort costs. These processes also interplay with serotonergic processes that promote reward learning and biasing people towards more cognitively effortful options (Westbrook, Bosch, Määttä, Hofmans, Papadopetraki, Cools, and Frank, 2020) even when it is suboptimal to deploy effort earlier - which could occur more often in people with low levels of short term working memory ability (Raghunath, Fournier, and Kogan, 2021)." Methods:

- The decision to utilize the rodent Cognitive Effort Task (rCET) without referencing or comparing it to other established measures like the Cognitive Effort Discounting Paradigm (COGED) represents a missed opportunity in the study's methodological rigor. This omission limits readers' understanding of the task's relative merits and suitability for investigating the research question. Providing a more thorough comparison or justification for the selection of the rCET could have enhanced the transparency and robustness of the study's methodology.

Response:

We appreciate this comment. We now further discuss our task in relation to existing human cognitive effort tasks. Specifically, we have more thoroughly justified why we used the rCET as a basis for our task in the Introduction section. On pages 7-8, we now discuss how the COG-ED task compares to existing tasks that inspired the rCET, such as the EEfRT task. We also discuss the advantages that rCET offers over COG-ED in terms of maintaining an incentive structure that participants must strategize within (instead of fully discounting easy and hard trial options) and maintaining translational comparability with rodent research.

- Moreover, the design choice of the study, contrasting only two levels of cognitive effort (easier vs. harder tasks) with directly associated rewards (low vs. high), has limitations compared to the parametric design employed in Cog-ED. First, a parametric design, offering multiple levels of effort and reward, would allow for a more nuanced understanding of how different degrees of effort influence decision-making. It can uncover dose-dependent relationships, providing richer insights into the dynamics between effort and reward.

Second, by always pairing high effort with high reward, and vice versa, the study's design conflates the effects of effort and reward sensitivity, making it difficult to disentangle which factor - effort or reward - more strongly influences a participant's choice. This limitation restricts the ability to fully understand the differential contributions of reward versus effort sensitivity in cognitive effort deployment.

Response:

We acknowledge that the design of our task only compares two effort levels and, thus, may offer less granularity of results than the COG-ED task. However, we did titrate the task difficulty based on participants' visual short term memory ability to match the task on difficulty between participants. Furthermore, in the interest of translational validity, we decided to keep the decision space between the rCET and our task the same. We discuss potential limitations of this approach in the Discussion section, as follows (pages 31-32): "Second, high effort trials never gave low rewards, and vice versa. This may impact our ability to evaluate whether it was sensitivity to effort or to reward that more strongly drove participant choices in the task. However, individual differences in reward anticipation - as measured in a task that involved accumulating did not predict choices for high effort deployment, suggesting that effort costs may be weighed more strongly than rewards by participants when making effort choices." "Furthermore, the choice structure of the task incorporated only two of the set sizes. This could have made the task excessively easy for some participants, and excessively diﬀicult to others. However, we did titrate task difficulty according to participants' visual short term memory ability, reducing the likelihood of the task difficulty not matching participant ability. Future studies could use a finer-grained, parametric design that offers choices at more diﬀiculty levels to examine whether motivation continuously varies with effort levels (Sayalı, Heling, and Cools, 2023)." - Why was the potential reward for high vs. low effort trials 10x greater even though the effort level was probably nowhere near 10x greater? Was it to greatly exaggerate the benefit-cost ratio for high effort trials relative to low effort trials, so that more people would choose high effort more often? Response:

As the reviewer noted, this effort ratio was set to incentivize participants to select high effort, high reward trials more often in the face of greater task difficulty. We have updated the methods section to reflect this page 14): "We set the high effort trial reward to 10 points to motivate participants to continue deploying effort even for a much more diﬀicult task." - Pg 11 line 224-225: Why was the N=8 condition not practiced although presented later? Is this a typo or a design choice? Please explain.

Response:

As the reviewer noted, the exclusion of the N=8 condition in the practice block was a design choice, so that participants could experience a subset of task conditions without becoming so experienced with high-difficulty task conditions that they would experience a ceiling effect on them. We have updated the methods section to reflect this (page 13): "Furthermore, although the main task had a maximum set size of 8, the practice block only included a maximum set size of 6 so as to give a brief overview of the trials and required responses without giving excessive practice with a large set size, for which performance would be evaluated in the subsequent block." - Lines 239-243: Would any results differ if the single-probe K estimate measure had been used instead of the whole-display K estimate measure? Response:

Although our task had a single probe, its position was randomized on each trial - requiring the participant to attend to the full display of shapes to identify which shape did or did not change. Upon reviewing this point, a single-probe K estimate measure - which is suitable when the position of the probe does not change - does not make sense. We now emphasize our use of the whole-display approach in the Methods section on pages 13-14, removing our mention of the single-probe measure and justifying the use of the whole-display measure. We also discuss the difference between these measures, and mention how results may differ with the single-probe approach, in the Discussion section on page 33: "Last, although our task only had a single probe whose colour had to be compared to the original probe, participants had to attend to the whole display to compare the single probe to the existing square at any given location on screen. As such, we used the whole-display K estimate measure to calibrate participant performance in our task (Rouder, Morey, Morey, and Cowan, 2011). A single-probe K estimate measure reflects lower effort demands than the whole-display measure, as the whole-display measure requires the status of all shapes on screen to be held in memory for the probe - whereas the single-probe measure only involves evaluating one shape in one position. However, as the position of the probe shape in our task is randomized, participants must still attend to the whole display to correctly identify any changes in the probe's colour. As such, the whole-display K estimate measure is more relevant for our task's structure and cognitive effort demands, and the results could differ if we used the single-probe measure." - Line 270 (and elsewhere): Why was the K estimate measure calculated based on the set size of 4? Response:

We calculated the K estimate measure based on a set size of 4 as, during piloting, this was determined to be the set size at which performance would drop off and the K estimate score would correspondingly decrease and become less representative of task performance. We have revised the Methods section to include additional information on this point (page 14): "This set size was determined through piloting to be the maximum set size before performance dropped off." - Pg 13. "Although participants were told that the number of points they gained in this phase was proportional to the monetary reward they would receive, all participants received the same reward" Instructing participants that the points gained would be proportional to the monetary reward, while ultimately providing the same reward to all, can introduce a bias towards selecting the harder task. This setup potentially confounds the study's aim to objectively measure cognitive effort deployment, as participants might be overly motivated by the prospect of a higher payout, skewing results towards high-effort choices irrespective of their natural inclination or capacity for effort. Such a design might not accurately reflect true effort-reward valuation, compromising the study's ability to distinguish between the intrinsic motivations for choosing different levels of cognitive effort. Moreover, individual differences in complying with instructions, or reward motivation (independent of effort) may also influence the findings. The authors are encouraged to visit the literature on human cognitive effort avoidance to review the format of their instructions and discuss how their instructions deviated from this literature.

Response:

We thank the reviewer for offering this important perspective. We acknowledge that our task was designed to incentivize participants to deploy more effort - especially as it was an online task where participant disengagement is more likely as compared to an in-person task. As the task was difficult to complete at larger set sizes (based on piloting), we anticipated - and determined through piloting - that participants would require a much larger number of points to be incentivized to choose harder trials. We still find that this strategy is useful for offering a more direct comparison to existing rodent (and human) cognitive effort tasks such as the rCET and COG-ED, as well as offering a straightforward task model that can be easily evaluated with approaches such as drift diffusion modelling. We also acknowledge that individual differences can exist in how participants interpret instructions and are motivated to seek reward. The instructions we have given were piloted and adapted from past studies where they were successful in guiding participant behaviour. However, we now note that in a future study, we could ask follow-up questions in which participants discuss their experiences of how valuable the reward was to them, and their compliance with the instructions. Such phenomenological context could help us better understand the participant experience in deploying varying levels of cognitive effort. We now further discuss these points in the Discussion section (pages 32-33): "Fifth, participants were told that the points they received would be proportional to the monetary reward received, which could motivate them towards higher effort choices regardless of their effort capabilities - shifting their effort-reward tradeoffs. However, such a strong effort incentive was necessary to motivate participant performance given the diﬀiculty of the high effort trials for many participants. Participants may have also individually differed in compliance with reading instructions or in their motivation for reward. Such factors could be investigated in a follow-up study incorporating qualitative evaluations of participant experiences of the task." Results:

- Pg 14. By classifying participants into just two categories based on a 70% cutoff for choosing the high-reward (HR) option, the study reduces the variability of participants' choices to a binary distinction, which may oversimplify the complex nature of cognitive effort deployment. This categorization overlooks the nuances and variability in individual decision-making processes, potentially masking subtler trends and relationships within the data. Analyzing choices as a continuous variable could provide a more detailed and nuanced understanding of how various factors influence the propensity to exert cognitive effort, offering richer insights into individual differences in effort-reward valuation.

Response:

We focused on the 70% cutoff (and, in general, a binary cutoff) to ensure comparability with existing rodent-based categorizations of motivation for high and low effort deployment. However, we acknowledge the importance of evaluating a continuous measure of effort choice preference. As such, we have added a linear model analysis predicting the proportion of high effort trials chosen, with visual short term memory ability (K score), depressive traits (BDI score), chronic stress (PSS score), and reward anticipation as predictors. We found that, once again, only visual short term memory ability significantly predicted the proportion of high effort trials selected. For our main analyses, we continue to focus on the binary distinction in order to predict participant classifications that map on to cognitive effort tasks in existing rodent research. We have updated the Methods section to reflect this analysis (page 17): "As choices for high-effort trials may also vary continuously with visual short term memory ability, we then conducted a linear regression to evaluate whether the above regressors also predict the proportion of high effort trials selected." We have also updated the Results section with the results of this analysis (pages 18-19): "Furthermore, a linear model analysis with the same predictors revealed that, once again, only visual short term memory (K estimate) scores significantly predicted the proportion of high effort trials that a participant selected (Table 3)." Additionally, we discuss our justification for the binarization of participant groups in the Methods section (page 16): "Although the grouping binarizes the outcomes of our analyses, it offers a clear point of translation from the rodent literature on which our task is based, and allows us to predict whether participants exhibit a high effort motivation vs. low effort motivation phenotype." - Lines 352-353: The fact that participants with higher working memory scores had a higher probability of being in the high effort group might simply be due to authors using K=3 as a binary cutoff to determine the effort levels that would be available for each participant to choose from in the choice task. But, some people might have been K=8 if the authors had allowed for that, which means that they would always choose high effort because it was actually easy for them than it would be for people with lower K scores. For example, maybe those participants who should have been classified as K=8 would have chosen fewer high effort trials if they were offered the choice between 6 boxes and 10 boxes.

Response:

We thank the reviewer for their input on this point. Our cutoff of K = 3 was, in fact, close to the maximum K estimate score that participants reached (K = 3.99). Given that participants' performance on high effort trials was not near to ceiling even if they were above the K estimate cutoff, we believe that, within the constraints of our task, participants would not likely be classified as K=8. Furthermore, given the maximum K score of 3.99, we cannot conduct an interpretable analysis of how the results might differ if the K estimate cutoff were higher.

- How correlated are the measures for depression, stress, reward anticipation, and working memory? Response:

We appreciate this question and now report the Pearson's r correlations among depressive traits (BDI score), chronic stress levels (PSS score), reward anticipation, and K estimate (working memory) in Figure 3 and in the Methods section (page 16): "As participants may express high levels of both depressive traits and chronic stress - potentially impacting reward anticipation (Alloy, Olino, Freed, and Nusslock, 2016) - we also evaluated the extent to which BDI scores, PSS scores, and reward anticipation were correlated with each other." We report the corresponding results in the Results section, as follows (page 18): "Levels of depressive traits (BDI), chronic stress (PSS), and reward anticipation were significantly correlated with each other; neither measure was significantly correlated with working memory ability (K estimate) (Fig. 3)." - Pg 18. Please report the actual values for accuracy / descriptive stats in a table or in text.

Response:

We now report descriptive statistics for accuracy and choice latency (mean and standard deviation) in Table 4, referenced on page 19 in the Results section.

- DDM Modeling: What is the evidence that is accumulating during the decision? DDM analysis usually relies on the theoretical foundation that there is clear evidence that is accumulating (e.g., physical motion energy for perceptual tasks or value estimation for preferential tasks). Here, it is not clear what the evidence would be. The author should try to offer a theoretical motivation for why a DDM should explain this sort of decision.

Response:

We thank the reviewer for their comments. We have added further discussion of how we interpret evidence accumulation in the context of selecting a higher effort trial, framing it as awareness of effort deployment abilities and motivation. We now discuss our interpretation of evidence accumulation in the introduction section (page 7): "In an equivalent task in humans, such evidence could include awareness of one's effort deployment willingness and ability as well as fatigue. For example, participants may build experience with how the task demands align with their short term memory capabilities, as well as their willingness to push these abilities further for higher reward. This evidence could also be used to make decisions about whether a higher reward is worth the additional effort." - Line 391: Is it sufficient to have only one trial of either LR or HR when fitting the DDM for a participant? Response:

We appreciate this point. As having only one trial of either HR and LR in the DDM may indeed not be sufficiently to accurately use that participant's behaviour in the DDM, we have revised our DDM fit to exclude participants who selected 3 or fewer high effort or low effort trials. These revised results are reported in the "Drift diffusion model" section of the Results on pages 23-24.

- General: Since all trials offer the same decision, why wouldn't a participant simply make the same choice on every trial? In other words, under the DDM framework, are all choices based purely on the diffusion noise combined with the starting point bias? Response:

As the reviewer indicated, choices in the DDM would be based primarily on diffusion noise and the starting point bias towards selecting a high effort trial. This bias could be impacted by fatigue and one's awareness of their effort deployment capability (in this case, likely driven by visual short term memory ability. The limited nature of this interpretation could suggest that the drift diffusion model did not capture meaningful, systematic patterns of effort deployment choices. We have added a discussion of these points in the Discussion section (pages 27-28): "Given that the decision available on each trial is the same, individual differences in this initial level of bias towards selecting high effort trials - together with random noise - is what drives differences in trial difficulty choice between participants as modeled by the drift diffusion model. Therefore, the drift diffusion model may not be capturing meaningful, systematic patterns of effort deployment choices. However, it still provides an exploratory evaluation of the underlying cognitive processes that could be driving effort deployment choices." Additionally, we have added further discussion of the theoretical rationale for the DDM in the Introduction section on pages 17-18 (discussed above).

- Overall results/discussion: The study's reliance on a binary task difficulty model (easy vs. hard) potentially obscures nuanced individual responses to cognitive effort. High working memory (WM) individuals performing almost at 100% on easier tasks suggests a mismatch between task demand and participant capability, possibly reflecting avoidance of boredom rather than effort. This is evidenced by High Effort participants' significant performance advantage in LR but not HR trials compared to Low Effort group and their initial bias against the easy task, which persists throughout the study. A parametric design, offering a gradient of difficulty levels, could better match tasks to individual abilities, avoiding ceiling effects and more accurately capturing the dynamics of motivation, effort, and enjoyment (Csikszentmihalyi's concept of flow, also covered in effort avoidance design in Sayalı, C., Heling, E., &Cools, R. (2023)). This approach would allow for a finer understanding of how cognitive capacity influences task preference beyond simplistic effort avoidance, emphasizing the importance of task engagement and its relation to cognitive abilities.

Sayalı, C., Heling, E., &Cools, R. (2023). Learning progress mediates the link between cognitive effort and task engagement. Cognition, 236, 105418.

Response:

We acknowledge that, although our binary task design is more in line with existing rodent cognitive effort tasks (e.g. rCET) and human tasks (e.g. EEfRT), a gradient of effort choices may more effectively parse out effects of boredom and ceiling effects in the task. However, as indicated above, we did titrate task difficulty based on participants' visual short term memory ability, which reduces the likelihood of trials being excessively easy or hard while maintaining a binary structure similar to that of the rCET. We discuss recommendations for future studies in the Discussion section (page 32): "Furthermore, the choice structure of the task incorporated only two of the set sizes. This could have made the task excessively easy for some participants, and excessively diﬀicult to others. However, we did titrate task difficulty according to participants' visual short term memory ability, reducing the likelihood of the task difficulty not matching participant ability. Future studies could use a finer-grained, parametric design that offers choices at more diﬀiculty levels to capture motivation and effort dynamics in a granular manner (Sayalı, Heling, and Cools, 2023)." - Discussion: The authors could revise the paper to more critically discuss the limitations of their current study design and interpretation of results. This includes acknowledging the potential for ceiling effects in easier tasks and the implications of using a binary classification system for effort choices. They could also explore the nuances of their findings in the context of existing human literature on effort and reward, including discussions on the potential for boredom or task engagement influencing choices, perhaps as a distinct factor that differentiates humans from rats. Future research directions could be proposed, highlighting the value of parametric designs and continuous measures for capturing a broader spectrum of cognitive effort deployment.

Response:

We thank the reviewer for this feedback. We have added a more critical evaluation of these limitations, as well as an additional discussion of future directions to address them, in the Discussion section (pages 33-34): "As many human cognitive effort tasks - unlike rodent tasks - do not require substantial training, a future study could examine effort choices given a wider range of effort and reward levels, as well as test how preference for effort changes given high effort trials with low reward and vice versa. Such a task could more richly capture individual differences in human cognitive effort deployment decisions." "Participant descriptions of their experiences in the task could indicate whether boredom and fatigue, in addition to reward motivation, impacted effort deployment choices. This approach would also allow us to further explore how rodent-based constructs of cognitive effort deployment are reflected in human decision-making, while extending our understanding of these constructs with human-specific experiential information." Minor comments - Figure 1 legend: Instead of "2 or 4, or 6 or 8" it should read "2 or 6, or 4 or 8" according to what is described in the text.

Response:

We thank the reviewer for their comment. We have corrected this typo in the Figure 1 legend.

- Line 272 (and elsewhere): It seems that the authors sometimes refer to "reward sensitivity" although at other times they refer to it as "reward anticipation". Are these the same thing? If so, I recommend using "anticipation" in all instances. "Sensitivity" gives the impression that they are better or worse at distinguishing between reward levels, although the authors clearly describe that they are interested in how motivating rewards are in general to an individual participant.

Response:

We thank the reviewer for their suggestion. We have been using "reward anticipation" and "reward sensitivity" to mean the same construct, related more closely to anticipation. As such, we have revised all mentions of "reward sensitivity" to "reward anticipation".

- Line 289: Perhaps "motivation" should be changed to something like "tendency".

Response:

We have replaced "motivation" with "tendency" on page 9.

- Line 317 (and elsewhere): Although the authors make it clear that the RT they examine is for the decision about which task to perform and not for the task itself, it may sometimes be confusing for the reader. This is because the authors mention RT in the same sentences or paragraphs as they mention accuracy, which does refer to the performance within the task itself. Perhaps they can find a way to rephrase the relevant sections.

Response:

We thank the reviewer for their suggestions. To clarify that reaction time refers to the choice latency for the choice and not for responding in the task itself, we have rephrased page 17 to read as follows: "We examined accuracy on each trial and choice latency for selecting the trial difficulty." - Line 366: "Group" should perhaps be replaced by "Trial Type".

Response:

We have replaced the word "Group" with "Trial Type" to correct the typo on page 19.

- Line 398: Perhaps remove the word "portion".

Response:

We have revised this sentence on page 20 to remove the word "portion".

- Line 407: The way it's phrased, this is not quite accurate. High effort participants needed more evidence before selecting the low effort trials, but not necessarily the high effort trials (because of the starting point bias).

Response:

We have revised this sentence on page 24 to read: "These findings suggest that participants who were in the high effort group required more evidence to select low but not high effort trials, ...".

- Lines 413-415: Doesn't this just show that working memory ability is continuous rather than binary? Response:

We interpret these results as support for working memory being the predominant driver of effort deployment decisions in this task. We expect that a parametric design with more effort levels would show a continuous gradient in terms of performance scaling with working memory ability. A parametric task design, which is now discussed as a potential future direction in the Discussion section, could give us a more precise indication of how working memory ability impacts effort deployment choices.

- Lines 417-419: Perhaps add that they required more evidence for low effort/reward trials.

Response:

We have revised pages 25-26 to add that: "Additionally, participants required more evidence to select LR trials compared to HR trials." - Figure 6: Please explain if boundary separation is from 0 to X or from -X to X. Then, please also explain what a starting point of 0.5 means with respect to X. For example, if boundary for a participant = 1.5, is the lower boundary 0 or -1.5... and is the starting point a percentage of the different between the boundaries or what? Response:We thank the reviewer for their recommendation. The boundary separation is from 0 to X, and the starting point is a percentage of the distance between the lower and upper boundaries. We have updated the Methods section on pages 17-18: "The starting point is expressed as a percentage of the distance between the lower and upper boundaries", and: "The boundary separation, expressed relative to 0, ...".

- Line 429: Perhaps "less" should read "more"? Response:

We say less as we interpret the increased performance on LR trials by participants who preferred HR trials as a reduced sensitivity to rewards and punishments, as they continued to select trials that they performed worse on and, overall, performing sub-optimally. To improve clarity, we have extended our interpretation of these results in the Discussion section (page 27): "Continually deploying high amounts of cognitive effort could be fatiguing; the high effort group's lower performance on HR trials could reflect the ineffectiveness of this high effort choice strategy." - Lines 438-439: Did reward sensitivity differ across groups? Response:

Reward anticipation did not significantly differ between the high and low effort preference groups. We have updated the discussion section to reflect this additional analysis on page 28: "These differences were not driven by reward anticipation, which did not differ between high and low effort groups (t(122.73) = 0.16, p = 1, d = 0.02)." - Line 439: The authors contrast their results against results from a previous study where there were correct and incorrect answers, but it's unclear how the concept of "correct" would apply in the current study.

Response: "Correct" refers to whether the subsequent response made on the chosen trial was correct or incorrect (as opposed to the choice of trial difficulty being modelled by the drift diffusion model). We have adjusted the text in the Discussion section on page 28 to clarify this point: "...while rodents in the equivalent high effort group had steeper drift rates when they subsequently made correct responses for their given choice (Hales, Silveira, Calderhead, Mortazavi, Hathaway, and Winstanley, 2024)." - Lines 443-445: This statement is not clear.

Response:

We have clarified this statement as follows (page 28): "Furthermore, given the conditionality of the drift rate findings in rodents, strategies driving impulsiveness or choice motivations to select a HR or LR trial could differ between humans and rodents." - Lines 454-457: The way it is phrased, it is not clear what the comparison is between when stating that certain rodents were "more accurate".

Response:

We have rephrased this statement to indicate that accuracy is in comparison to the other effort trial type, and that accuracy is for on the trial itself and not for the choice of the trial effort level. We have updated the Discussion section (page 29) as follows: "...showed that rodents choosing more HR trials were slightly more accurate on HR than LR trials when completing the trials themselves, while rodents choosing more LR trials were slightly more accurate on LR than HR trials when completing these trials." - Lines 489-490: But why would this vary across trials? Response:

These effort trade-offs could shift across the task as participants become fatigued or bored with the task, or if they determine a more effective strategy that shifts how they choose to deploy effort. We now discuss this in the Discussion section, page 27: "These tradeoffs could shift over time in the task as participants become fatigued or bored with the task, or find a new effort deployment strategy that better optimized rewards or task enjoyment".

- Lines 497-501: It seems that such an explanation would require the authors to include in their analysis a measure of performance on trials prior to the current trial.

- This paragraph was hard to follow. Furthermore, it seems that this sort of explanation should be mentioned earlier.

Response:

We have now conducted an additional linear regression analysis evaluating whether performance on the previous trial predicts performance on the current trial, in interaction with whether participants chose more high effort trials. Although prior trial performance significantly predicted current trial performance, this prediction did not differ by effort preference group. We now report these findings in the Discussion section, pages 29-30: "We conducted a follow-up linear regression analysis to evaluate whether performance on the previous trial predicts performance on the current trial as a function of effort motivation group and trial type. Performance on the prior trial, but not effort motivation group, significantly predicted performance on the current trial (t(68955) = 8.45, p = <0.001), suggesting that, overall, participants maintained a constant performance-based strategy that was not explained by effort choice decisions." Furthermore, we have split up and restructured this paragraph to bring the discussion of choice latency earlier in the discussion and to improve overall clarity. This first part of the paragraph is now on page 27.

- There are several very minor typos in the manuscript, so a solid proofing for such minor typos would be advised.

Response:

We and have carefully proofread the paper to correct typos.
